# Neural network assisted Kalman filter for INS/UWB integrated seamless quadrotor localization

**DOI:** 10.7717/peerj-cs.630

**Published:** 2021-07-14

**Authors:** Shuhui Bi, Liyao Ma, Tao Shen, Yuan Xu, Fukun Li

**Affiliations:** School of Electrical Engineering, University of Jinan, Jinan, Shandong, China

**Keywords:** Neural network assisted Kalman filter, INS/UWB, Quadrotor, Localization

## Abstract

Due to some harsh indoor environments, the signal of the ultra wide band (UWB) may be lost, which makes the data fusion filter can not work. For overcoming this problem, the neural network (NN) assisted Kalman filter (KF) for fusing the UWB and the inertial navigation system (INS) data seamlessly is present in this work. In this approach, when the UWB data is available, both the UWB and the INS are able to provide the position information of the quadrotor, and thus, the KF is used to provide the localization information by the fusion of position difference between the INS and the UWB, meanwhile, the KF can provide the estimation of the INS position error, which is able to assist the NN to build the mapping between the state vector and the measurement vector off-line. The NN can estimate the KF’s measurement when the UWB data is unavailable. For confirming the effectiveness of the proposed method, one real test has been done. The test’s results demonstrate that the proposed NN assisted KF is effective to the fusion of INS and UWB data seamlessly, which shows obvious improvement of localization accuracy. Compared with the LS-SVM assisted KF, the proposed NN assisted KF is able to reduce the localization error by about 54.34%.

## Introduction

Nowadays, the quadrotor has been widely used in many fields (Xu et al., 2020a; Nguyen & Hong, 2019; Kou et al., 2018). Consequently, many approaches have been proposed for the quadrotor (Liang et al., 2019). In order to make the quadrotor have better performance, the accurate localization scheme, which is the key technology of the quadrotor to accomplish other tasks, should be investigated (Camci & Kayacan, 2019).

To the localization technologies for the quadrotor, there are many approaches have been proposed. For instance, a smart quadcopter aircraft navigation system using the global positioning system (GPS) was designed, which can achieve autonomous flight control with smooth and stable maneuvering, see Bonny & Abdelsalam (2019). Global navigation satellite systems (GNSS) intigrating light detection and ranging (LiDAR) scheme was investigated to achieve the autonomous navigation in forests (Chiella et al., 2019). The indoor quadrotor localization integrated by inertial navigation system (INS) and ultra wide band (UWB) was proposed by Xu et al. (2020b). A high-speed autonomous quadrotor navigation through visual and inertial paths was proposed (Do, Carrillo-Arce & Roumeliotis, 2019). Autonomous vision-based micro air vehicle for indoor and outdoor navigation was investigated in Schmid et al. (2014). It should be emphasized that the basic idea of the approaches mentioned above is to replace the unavailable positioning technology with a available one.

In aggregate, the data fusion filter has played an important role in integrated navigation system (Zhao & Huang, 2020; Wang et al., 2018; Li et al., 2019; Liu, Yu & Shuang, 2019). Moreover, the Kalman filter (KF) with its improving filters have been proposed for the data fusion (Liu et al., 2020). For example, the fading cubature Kalman filter (CKF) was designed to the initial alignment of strapdown inertial navigation system (SINS) (Guo et al., 2020). The quadrotor state estimation based on CKF was proposed (Benzerrouk, Nebylov & Salhi, 2016). An improving CKF method was investigated for the the attitude determination system of missile (Liu et al., 2019). The CKF is used for the GNSS/INS under GNSS-challenged environment (Cui et al., 2019). An improved square root unscented Kalman filter was proposed for the localization of the coaxial Quadrotor (Gośliński et al., 2019). A Kalman filter/expectation maximization (EM) integrated frame was proposed in Qin et al. (2020). A new approach for enhancing the indoor navigation of unmanned aerial vehicles (UAVs) with velocity update applied to an extended Kalman filter (EKF) was investigated by Zahran et al. (2019). It should be pointed out that the outage of the data fusion filter’s measurement are not considered by the approaches mentioned above. Meanwhile, in order to ensure that the data fusion filter works, some artificial intelligence (AI)-based methods have been proposed, which have been used used in other fields (Zhang et al., 2021, 2020).

In this paper, we propose a neural network (NN) assisted KF, which is able to deal with the missing data in case of UWB data outage. Neural network is used to build the mapping between states and observations. The performance is verified with real data. Comparison shows that the proposed approach outperforms LS-SVM algorithm significantly in accuracy improvement.

The contributions of this work are listed in the following:

- A new NN assisted KF for fusing the UWB and INS data seamlessly is presented in this work, which employs the NN to build mapping between states and observations offline and predict the observations when the UWB is outage.
- Real tests have been done for demonstrating the effectiveness of the proposed approach.

The remainder structure of this article is sketched as follows. The description of INS/UWB integrated seamless quadrotor localization scheme is given in “INS/UWB Integrated Seamless Quadrotor Localization Scheme”. “Kalman Flilter” and “The Scheme of the NN” investigated the KF and the NN method for the localization scheme of INS/UWB integrated seamless quadrotor. The test is done in the “Test” section. Finally, conclusions are drawn in the “Conclusion” section.

## INS/UWB Integrated Seamless Quadrotor Localization Scheme

**Figure 1:**
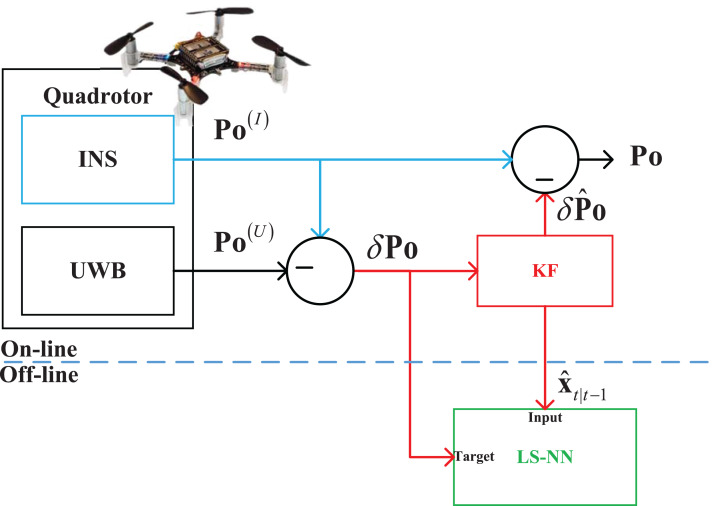
The data fusion scheme when the UWB measurements are available.

**Figure 2:**
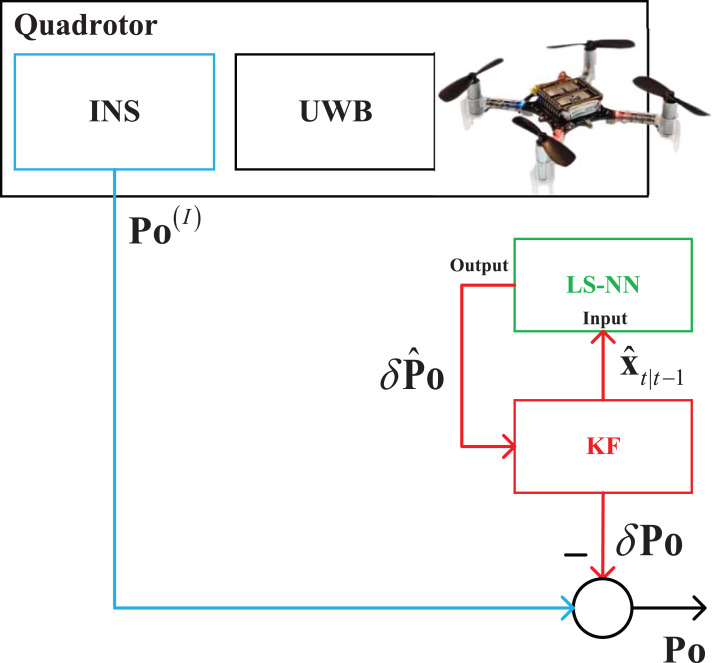
The data fusion scheme when the UWB measurements are unavailable.

In this section, the INS/UWB integrated seamless quadrotor localization scheme will be designed in two cases. The integrated seamless scheme proposed in this work are listed in the following:

- When the UWB measurements are available, the data fusion scheme is shown in Fig. 1. In this situation, the INS and UWB localization technologies measure the target quadrotor’s position **Po**^(I)^ and **Po**^(U)^ respectively. Then, the Kalman filter (KF) estimates the position **Po** by fusing the **Po**^(I)^ and **Po**^(U)^.
- Using the outputs and the measurements of the KF when the UWB measurements are available, the NN works in the training stage, it builds the mapping between the KF’s measurement *δ***Po**_*t*_, *t* ∈ [1, +∞) and the data filter’s state vector }{}{\hat x_{t|t - 1}},t \in [1, + \infty ) after normal flight of the quadrotor. Here, the *t* is the time index. It should be pointed out that both the *δ***Po**_*t*_, *t* ∈ [1, ∞) and the }{}{\hat x_{t|t - 1}},t \in [1, \infty ) are collected when the KF works normally, and the building process of the mapping is off-line.
- When the UWB measurements are not available, the data fusion scheme can be designed as Fig. 2. In this situation, the UWB is unable to provide the **Po**^(U)^ due to the outage of the UWB. Thus, the KF is unable to work. In this situation, the NN is employed to rebuild the measurement of the KF. It works in prediction stage, which is utilized to provide the estimated position error *δ***Po** by using the mapping built in the above stage and the }{}{\hat x_{t|t - 1}}. Then, the *δ***Po** is used as the measurement of the KF, which makes the KF can work when the UWB measurement is outage.

## Kalman Filter

Based on the seamless integrated scheme, the KF used in this work will be introduced in this section. The state equation of KF used in this work is listed in Eq. (1).

(1) \underbrace {\left[ {\matrix{ {\delta {\bf{P}}{{\bf{o}}_{t|t - 1}}} \cr {\delta {{\bf{V}}_{t|t - 1}}} \cr } } \right]}_{{{\bf{x}}_{t|t - 1}}} = \underbrace {\left[ {\matrix{ {{{\bf{I}}_{3 \times 3}}} & {\delta t\cdot{{\bf{I}}_{3 \times 3}}} \cr {{{\bf{0}}_{3 \times 3}}} & {{{\bf{I}}_{3 \times 3}}} \cr } } \right]}_{\bf{F}}\underbrace {\left[ {\matrix{ {\delta {\bf{P}}{{\bf{o}}_{t - 1}}} \cr {\delta {{\bf{V}}_{t - 1}}} \cr } } \right]}_{{{\bf{x}}_{t - 1}}} + {{\bf{\omega }}_{t - 1}}

where the time index is denoted as *t*, *δt* means the sample time, \delta {{\bf Po}_t} = {\left[ {\delta {x_t},\delta {y_t},\delta {z_t}} \right]^T} means the position error vector at the time index *t*, here, the \left( {\delta {x_t},\delta {y_t},\delta {z_t}} \right) means the position error in the east, north, and up direction respectively, \delta {{\bf V}_t} = {\left[ {\delta V{x_t},\delta V{y_t},\delta V{z_t}} \right]^T} means the velocity error vector at the time index *t*, here, the \left( {\delta V{x_t},\delta V{y_t},\delta V{z_t}} \right) means the velocity error in the east, north, and up direction respectively, *ω*_*t*−1_ ∼ N(0, Q) is the system noise and Q is its covariance.

The measurement equation of KF used in this work is listed in Eq. (2).

(2) \underbrace {\left[ {\matrix{ {x_t^{\left( I \right)} - x_t^{\left( U \right)}} \cr {y_t^{\left( I \right)} - y_t^{\left( U \right)}} \cr {z_t^{\left( I \right)} - z_t^{\left( U \right)}} \cr } } \right]}_{{{\bf{Y}}_t}} = \underbrace {\left[ {\matrix{ {{{\bf{I}}_{3 \times 3}}} & {{{\bf{0}}_{3 \times 3}}} \cr } } \right]}_{\bf{H}}{{\bf{x}}_{t|t - 1}} + {{{\upsilon }}_{t - 1}},

where }{}( {x_t^{\left( I \right)},y_t^{\left( I \right)},z_t^{\left( I \right)}} \right) is the INS-measured position **Po**^(*I*)^ in east, north, and the upside direction, respectively, }{}( {x_t^{( U )},y_t^{( U \right)},z_t^{\left( U \right)}} \right) is the UWB-measured position **Po**^(*U*)^ in east, north, and the up direction respectively, *υ*_*t*_ ∼ N(0, R) is the measurement noise and R is its covariance. The KF filtering algorithm based on the model (1) and (2) is listed in Algorithm 1.

**Algorithm 1:** The KF filtering algorithm based on the model (1) and (2).

**Data**: **Y**_*t*_, **Q**, **R**
**Result:** }{}{\hat {\bf x}_t},}{}{\hat {\bf P}_t}
1 **begin**
2 **for** *t* = 1: ∞ **do**
3 }{}{\hat {\bf x}_{t|t - 1}} = {\bf F}{\hat {\bf x}_{t - 1}};
4 }{}{\hat {\bf P}_{t|t - 1}} = {\bf F}{\hat {\bf P}_{t - 1}}{{\bf F}^T} + {\bf Q};
5 }{}{{\bf K}_t} = {\hat {\bf P}_{t|t - 1}}{{\bf H}^T}{\left( {{\bf H}{{\hat {\bf P}}_{t|t - 1}}{{\bf H}^T} + {\bf R}} \right)^{ - 1}};
6 }{}{\hat {\bf x}_t} = {\hat {\bf x}_{t|t - 1}} + {{\bf K}_t}\left[ {{{\bf Y}_t} - H{{\hat {\bf x}}_{t|t - 1}}} \right];
7 }{}{\hat {\bf P}_t} = \left( {{\bf I} - {{\bf K}_t}{\bf H}} \right){\hat {\bf P}_{t|t - 1}};
8 **end for**
9 **end**

## The Scheme of the Neural Network (NN)

In case of outage in complex indoor environment, due to the lack of UWB measurements, the observation vector in Kalman filter become unavailable. To provide the observation vector for the data fusion filter, the Neural Network (NN) is employed in this work.

However, it should be noticed that it is hard to model mathematically the relation between the measurements of the data fusion filter **Y**_*t*_ and the state vector }{}{\hat {\bf x}_{t|t - 1}}. For overcoming this issue, the NN is trained to build the mapping between them using the KF’s measurement **Y**_*t*_, *t* ∈ [1, +∞) and the }{}{\hat {\bf x}_{t|t - 1}},t \in [1, + \infty ) collected after normal flight of the quadrotor. The input and target of the NN model are chosen as }{}{\hat {\bf x}_{t|t - 1}} and **Y**_*t*_ respectively. In this work, we select the simple BP neural network structure without hidden layer. Build the mapping between }{}{\hat {\bf x}_{t|t - 1}} and **Y**_*t*_ using the *δ***Po**_*t*_, *t*∈ [1,∞) and the }{}{\hat {\bf x}_{t|t - 1}},t \in [1,\infty ) via NN.

The NN method is summarised in Algorithms 2 and 3. In the Algorithm 2, the KF provides the }{}{\hat {\bf x}_t} and the }{}{\hat {\bf P}_t} normally. Then, the NN is used to build the mapping between }{}{\hat {\bf x}_{t|t - 1}} and **Y**_*t*_ using the *δ***Po**_*t*_, *t* ∈ [1,∞) and the }{}{\hat {\bf x}_{t|t - 1}},t \in [1,\infty ) on off-line model.

**Algorithm 2:** NN assisted Kalman filtering algorithm (off-line model).

**Data**: **Y**_*t*_, **Q**, **R**
**Result:** }{}{\hat {\bf x}_t},}{}{\hat {\bf P}_t} the mapping between }{}{\hat {\bf x}_t}_|t −1_ and **Y**_t_
1 **begin**
2 **for** *`t* = 1:*∞* **do**
3 }{}{\hat {\bf x}_{t|t - 1}} = {\bf F}{\hat {\bf x}_{t - 1}};
4 }{}{\hat {\bf P}_{t|t - 1}} = {\bf F}{\hat {\bf P}_{t - 1}}{{\bf F}^T} + {\bf Q};
5 }{}{{\bf K}_t} = {\hat {\bf P}_{t|t - 1}}{{\bf H}^T}{\left( {{\bf H}{{\hat {\bf P}}_{t|t - 1}}{{\bf H}^T} + {\bf R}} \right)^{ - 1}};
6 }{}{\hat {\bf x}_t} = {\hat {\bf x}_{t|t - 1}} + {{\bf K}_t}\left[ {{{\bf Y}_t} - H{{\hat {\bf x}}_{t|t - 1}}} \right];
7 }{}{\hat {\bf P}_t} = \left( {{\bf I} - {{\bf K}_t}{\bf H}} \right){\hat {\bf P}_{t|t - 1}};
8 **end for**
9 Build the mapping between }{}{\hat {\bf x}_{t|t - 1}} and **Y**_*t*_ using the *δ***Po**_*t*_, *t* ∈ [1,*∞*) and the }{}{\hat {\bf x}_{t|t - 1}},t \in [1,\infty ) via NN;
10 **end**

**Algorithm 3:** NN assisted Kalman filtering algorithm (on-line model).

**Data**: **Y**_*t*_, **Q**, **R**, the mapping between }{}{\hat {\bf x}_{t|t - 1}} and **Y**_*t*_
**Result:** }{}{\hat {\bf x}_t},}{}{\hat {\bf P}_t}
1 **begin**
2 **for** *t* = 1:*∞* **do**
3 }{}{\hat {\bf x}_{t|t - 1}} = {\bf F}{\hat {\bf x}_{t - 1}};
4 }{}{\hat {\bf P}_{t|t - 1}} = {\bf F}{\hat {\bf P}_{t - 1}}{{\bf F}^T} + {\bf Q};
5 **if** **Po**^(U)^ is *available* **then**
6 }{}{{\bf Y}_t} = \left[ {\matrix{ {x_t^{\left( I \right)} - x_t^{\left( U \right)}} \cr {y_t^{\left( I \right)} - y_t^{\left( U \right)}} \cr {z_t^{\left( I \right)} - z_t^{\left( U \right)}} } } \right];
7 **else**
8 Estimate **Y**_*t*_ using }{}{\hat {\bf x}_{t|t - 1}} and previously built the mapping between }{}{\hat {\bf x}_{t|t - 1}} and **Y**_*t*_ via NN;
9 **end if**
10 }{}{{\bf K}_t} = {\hat {\bf P}_{t|t - 1}}{{\bf H}^T}{\left( {{\bf H}{{\hat {\bf P}}_{t|t - 1}}{{\bf H}^T} + {\bf R}} \right)^{ - 1}};
11 }{}{\hat {\bf x}_t} = {\hat {\bf x}_{t|t - 1}} + {{\bf K}_t}\left[ {{{\bf Y}_t} - {\bf H}{{\hat {\bf x}}_{{\bf t|t - 1}}}} \right];
12 }{}{\hat {\bf P}_t} = \left( {{\bf I} - {{\bf K}_t}{\bf H}} \right){\hat {\bf P}_{t|t - 1}};
13 **end for**
14 **end**

In the Algorithm 3, the KF works normally when the **Po**^(U)^ is available. Here, the KF is used to provide the estimation of the *δ***Po** using the observation vector }{}{\bf Y_t} = {\left[ {\matrix{ {x_t^{{\rm (I)}} - x_t^{{\rm (U)}}} & {{\rm y}_t^{{\rm (I)}} - y_t^{{\rm (U)}}} & {z_t^{{\rm (I)}} - z_t^{{\rm (U)}}}} } \right]^T}. Once the **Po**^(U)^ is unavailable, the proposed NN assisted Kalman filtering algorithm estimate **Y**_*t*_ using }{}{\hat {\bf x}_{t|t - 1}} and previously built mapping via NN.

## Test

In order to demonstrate the effectiveness of the proposed method, the real test will be investigated in this section.

### Experimental settings

**Figure 3:**
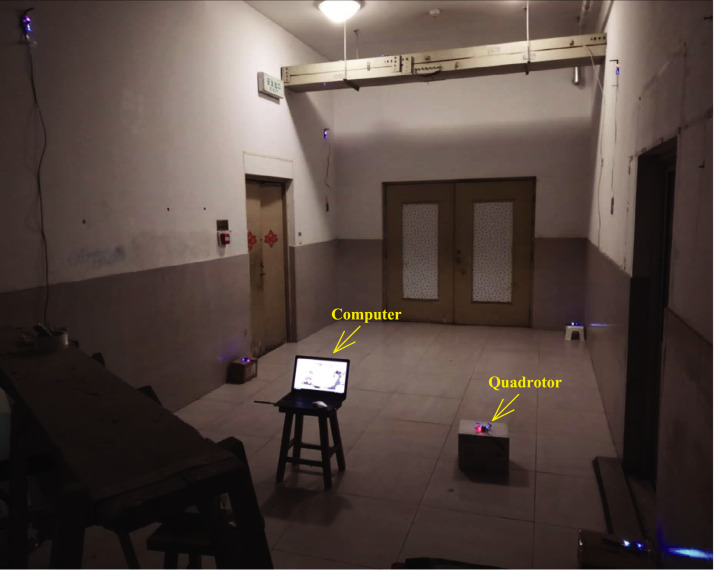
Test environment.

**Figure 4:**
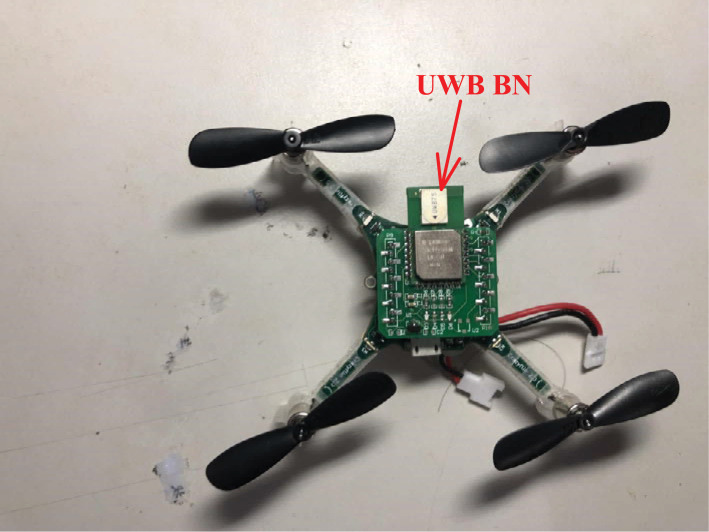
The quadrotor used in this work.

**Figure 5:**
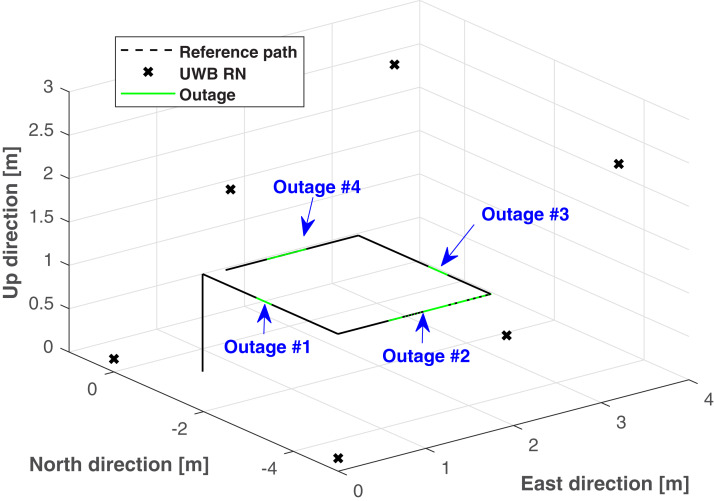
The reference path, UWB RNs, and the outage areas used in the test.

In this section, the real test will be considered to show the validity of the proposed method. The real test is done in the No. 1 building, University of Jinan, China, the test environment is displayed in Fig. 3. The quadrotor used in this work is shown in Fig. 4. Here, we employ the quadrotor to carry UWB blind node (BN) and the inertial measurement unit (IMU). The UWB BN fixed on the target quadrotor is able to collect the distances *d*_*i*_,*i* ∈ [1, 6] between the target quadrotor and the UWB reference node (RN). Here, the *i* has the same number as the UWB RN. Then, the UWB position **Po**^(U)^ can be computed via the the *d*_*i*_,*i* ∈ [1, 6]. And the INS position **Po**^(I)^ is provided by the IMU. The difference *δ***Po** between the **Po**^(I)^ and **Po**^(U)^ is used as the measurement of the KF. In the test, the quadrotor runs following the reference path, which is shown in Fig. 5. In this work, the sample time is set to 0.02*s*. In order to indicate the effect of the proposed method, four UWB outage areas (#1, #2, #3, and #4) are simulated as shown in Fig. 5.

### Localization errors

In this subsection, the performance of the proposed NN assisted KF will be investigated. Here, we compare the NN assisted KF’s performance with the least squares support vector machine (LS-SVM) assisted KF. In this work, we employ the mean square error (MSE) at each time index, which is calculated by the follows:

(3) \matrix{ {{\rm MSE}{{\left( {{\bf Po}} \right)}_t}} \cr { = \displaystyle{1 \over 3}\left( {{{\left( {{x_t} - x_t^{ref}} \right)}^2} + {{\left( {{y_t} - y_t^{ref}} \right)}^2} + {{\left( {{z_t} - z_t^{ref}} \right)}^2}} \right)} \cr {} \cr } {\kern 1pt} ,

where }{}{\rm MSE}{\left( {{\bf Po}} \right)_t} means the MSE of the position at time index *t*, \left( {{x_t},{y_t},{z_t}} \right) is the estimated position in *x*, *y*, and *z* directions at the time index *t*, \left( {x_t^{ref},y_t^{ref},z_t^{ref}} \right) is the reference position in *x*, *y*, and *z* directions at the time index *t*.

**Figure 6:**
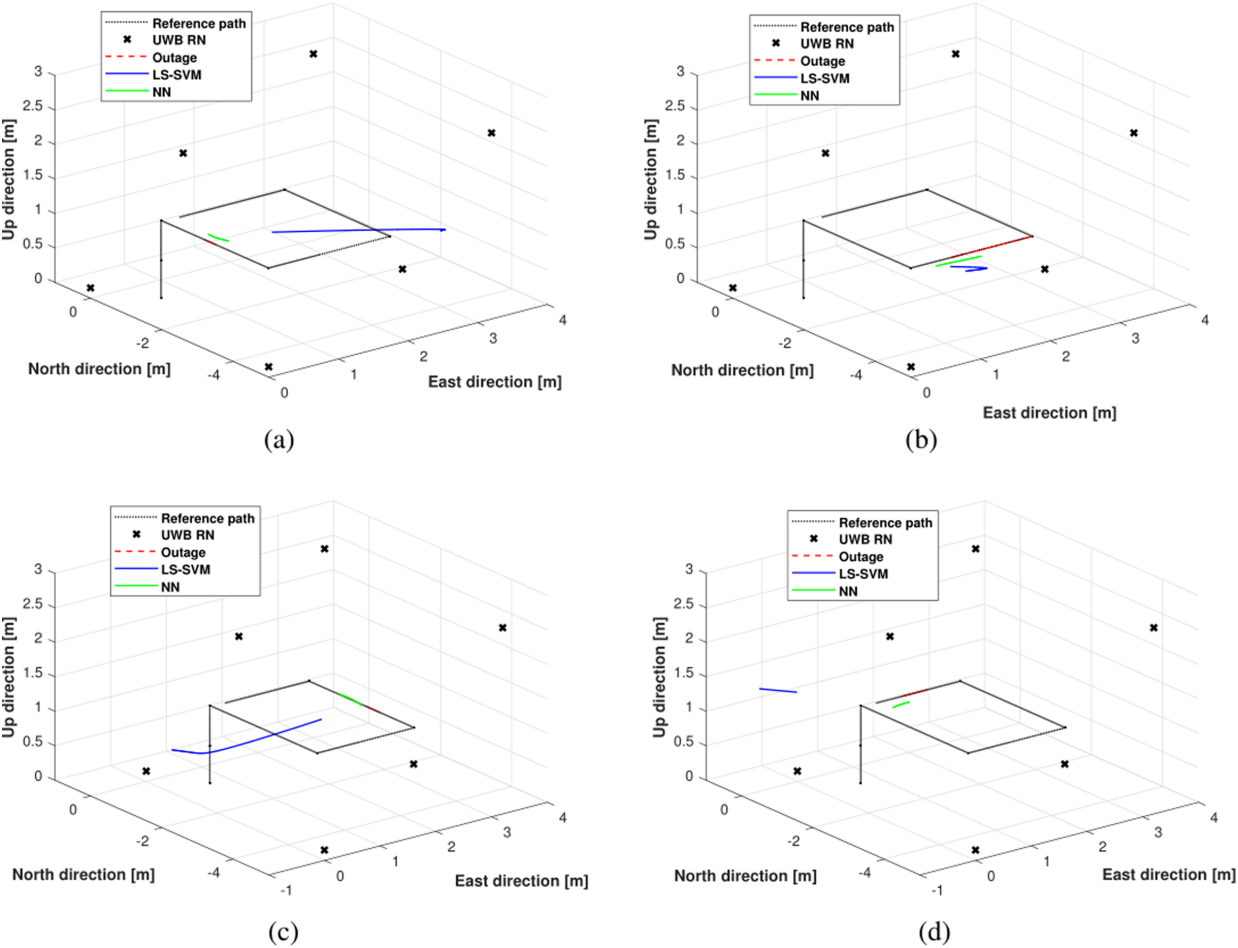
The trajectories estimated by the LS-SVM and the NN in outage areas: (A) outage #1, (B) outage #2, (C) outage #3, and (D) outage #4.

Figure 6 shows the trajectories estimated by the LS-SVM and the NN in outage areas #1, #2, #3, and #4. From the figures, one can see easily that in the outages areas #1, #2, #3, and #4, when UWB measurements are unavailable, the NN can still make decisions that are close to the reference path, while the LS-SVM algorithm gives a large accumulated error.

**Figure 7:**
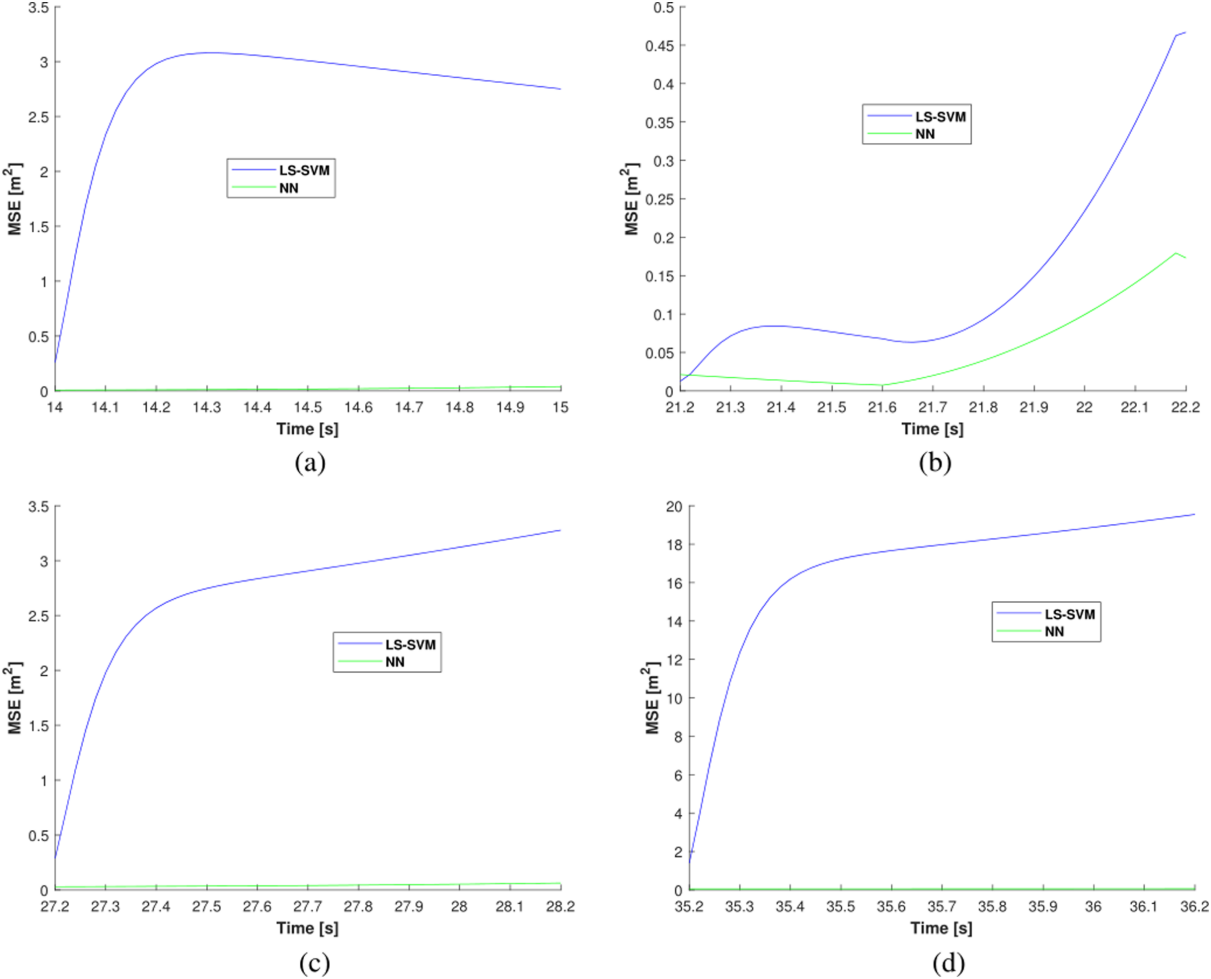
The MSEs estimated by the LS-SVM and the NN in outage areas: (A) outage #1, (B) outage #2, (C) outage #3, and (D) outage #4.

The MSEs estimated by NN (green line) and LS-SVM (blue line) in the outages areas #1, #2, #3, and #4 are shown in Fig. 7. From the figures, one can see that the MSE of the LS-SVM algorithm has a larger accumulated error compared with the NN. The average MSEs Produced by NN and LS-SVM in the outages areas #1, #2, #3, and #4 are listed in Table 1. It can be infered from the table that the average MSEs of the NN are smaller than the LS-SVM in the outages areas #1, #2, #3, and #4. Compared with the LS-SVM, the proposed NN reduced the localization error by about 54.34%. Thus, we can conclude that the proposed NN-based method can effectively reduce the localization error.

**Table 1:** Average MSEs produced by NN and LS-SVM in outages #1–#4.

Method	MSE (*m*^2^)				
	#1	#2	#3	#4	Mean
LS-SVM	2.7445	0.1453	2.7147	16.6635	5.5670
NN	0.0190	0.0524	0.0422	0.0537	2.5418

## Conclusion

In this work, in order to make the data fusion filter work properly under the condition that the UWB data is unavailable due to some harsh indoor environments, the NN assisted KF for fusing the UWB and the INS data seamlessly has be investigated. The contributions of this work are summarized as following:

- An NN assisted KF scheme has been designed for fusing the INS and UWB measurement.
- The model of the KF for the integrated scheme has been investigated.
- The NN assisted KF for fusing the UWB and the INS data seamlessly has been investigated. In the proposed approach, the KF provides the localization information when the UWB data is available. Meanwhile, the KF is used to assist the NN to build the mapping between the }{}{\hat {\bf x}_{t|t - 1}} and **Y**_*t*_ off-line. The NN can estimate the measurement vector of the KF when the UWB data is unavailable.
- Real tests have been done to show better performance of the proposed approach.

Based on the results presented in this work, we are now working on further developments of the proposed algorithms to build the mapping with the deep learning and plan to report the results in the near future.

## Supplemental Information

10.7717/peerj-cs.630/supp-1 Supplemental Information 1 Distance information measured by UWB.

10.7717/peerj-cs.630/supp-2 Supplemental Information 2 Location information measured by INS.

10.7717/peerj-cs.630/supp-3 Supplemental Information 3 Reference trajectory.

10.7717/peerj-cs.630/supp-4 Supplemental Information 4 Heading information.

10.7717/peerj-cs.630/supp-5 Supplemental Information 5 KF based estimation procedure.

10.7717/peerj-cs.630/supp-6 Supplemental Information 6 KF based prediction program.

10.7717/peerj-cs.630/supp-7 Supplemental Information 7 Main simulation program.

## Additional Information and Declarations

### Data Availability

The following information was supplied regarding data availability: The raw data and codes are available in the Supplemental Files.
